# SmUDo (Smart Unit-Dose): Redefining efficiency, quality, and staffing strategies for optimized processes

**DOI:** 10.1371/journal.pone.0339381

**Published:** 2026-01-16

**Authors:** Jana Gerstmeier, Saskia Herrmann, Annika Demuth, Natalie Vuong, Olaf Kannt, Dominic Fenske

**Affiliations:** 1 Hospital Pharmacy, Helios Kliniken GmbH, Berlin, Germany; 2 Department of Pharmaceutical/Medicinal Chemistry, Institute of Pharmacy, Friedrich Schiller University Jena, Jena, Thuringia, Germany; 3 Clinical Pharmacy, Helios Klinikum Erfurt, Campus of the Health and Medical University Erfurt, Thuringia, Germany; 4 Medical Management, Helios Kliniken GmbH, Berlin, Germany; 5 Pharmacy Department, University Medical Center, Johannes Gutenberg-University Mainz, Mainz, Germany; Florida International University, UNITED STATES OF AMERICA

## Abstract

**Background:**

The implementation of unit-dose dispensing systems (UDDS) in hospital pharmacies represents a major milestone in the digital transformation of medication therapy management, operational efficiency, and improving patient safety. However, the absence of standardized metrics for workforce planning and cost assessment has limited widespread adoption. This gap makes it increasingly challenging for healthcare stakeholders to optimize the allocation of limited medical and financial resources.

**Methods:**

Using data from a 1,800-bed tertiary care hospital with 1,680 UDDS beds (2021–2024), this study establishes benchmarks for staffing requirements and associated costs. The following key performance metrics were analyzed: task-specific work times, unit-dose machine utilization, and productivity indicators such as blistered tablet output and blister bag production per full-time equivalent (FTE).

**Results:**

The findings indicate a staffing benchmark of 0.45 FTE per 100 UDDS-covered hospital beds to support both implementation and routine operations. Workforce efficiency improved significantly, with the number of FTEs per 1 million blistered tablets decreasing from 3.2 in 2021 to 1.7 in 2024. Concurrently, the cost per blister bag improved from €0.55 in 2021 to €0.22 in 2024, well within the optimal unit target price range of €0.20-€0.25; blistered tablet production tripled, rising from 1.5x10^6^ to 4.7x10^6^ between 2021 and 2024 respectively. A gold standard of 1 FTE per 10,000 cases was established to optimize staffing and operational workflows. The sharp decline in FTE per 1 million requirements from 5.45 to 2.29 underscores the pivotal role of experienced clinical pharmacists in ensuring safe, accurate, and resource-optimized prescription validation.

**Conclusion:**

The proposed benchmarks provide practical guidance for improving UDDS productivity, optimizing resource allocation, and strengthening workforce management. Our findings provide a framework that supports continuous UDDS improvement in hospital pharmacy operations and evidence-based decision-making for key stakeholders. By establishing clear UDDS benchmarks, this study supports cost-efficient UDDS operations while providing surrogate indicators of patient safety and clinical quality.

## Introduction

Medication errors remain a widespread and significant problem within healthcare systems often resulting in preventable harm and adverse patient outcomes. In response to this persistent challenge, the World Health Organization (WHO) launched the “Medication Without Harm” initiative to improve medication safety on a global scale [1,2]. The goal is simple but ambitious. Safe medication management requires a proactive approach such as the implementation of unit-dose dispensing systems (UDDS). These robotic-based systems involve packaging individual doses of medication, labeled with critical information to ensure both the administered medication and patient are correctly identified [3]. UDDS not only enhances patient safety, it also delivers substantial economic and staffing benefits [3].

Although these technologies require initial investments, they yield a substantial return on investment (ROI) in the long term, as other automated dispensing systems used in clinical pharmacies proved years ago [4–7]. The following key stages of the medication process are particularly prone to errors: prescribing, dispensing, administering, monitoring, and documenting. Decreased error rates can lower costs associated with adverse drug events (ADE) while efficient workflow management further contributes to financial savings [8]. Digital health technologies are increasingly recognized as vital tools in reducing those risks [9]; indeed, governments worldwide have been embracing digital health solutions to enhance patient safety. In 2010, the United States introduced the Health Information Technology for Economic and Clinical Health (HITECH) Act to promote the widespread adoption of Electronic Health Records (EHRs) [10]. The United Kingdom’s National Health Service (NHS) focuses on digital-first primary care and optimizing hospital operations through programs like the “Hospital Pharmacy Transformation Programme” (HPTP) [11]. HPTP’s principal objective is to improve services by increasing the number of pharmacist prescribers via the implementation of e-prescribing and administration. Such initiatives aim to enable clinical pharmacists (CPs) and clinical pharmaceutical technical assistants (PTAs) to spend more time on patient-facing medicine optimization [11]. Similarly, Germany’s 2020 Hospital Futures Act mandates hospital infrastructure digitalization and emphasizes medication management as a key component for improving patient care. As hospitals are increasingly evaluated based on their digital maturity, a digital medication management system, combined with a UDDS as the gold standard, is essential [12]. Collectively, those global initiatives are aimed at improving patient outcomes and optimizing workforce efficiency, and highlight the broader trend toward digital transformation in healthcare [13].

A critical element of digital prescribing is the use of clinical decision support systems (CDSS) that have been shown to reduce prescription errors (PE); these provide real-time alerts for drug interactions, overdoses, and allergies at the point of prescribing [13,14]. In particular, CDSS have driven a paradigm shift in healthcare since their introduction over 50 years ago [15]. Moreover, a modern electronic medication system (EMS) ensures the “6 Rights” of medication administration: right patient, medication, dose, time, route of application, and documentation [16,17]. A fully digitized EMS, referred to as closed-loop medication management (CLMM), covers the entire medication process from prescription through administration [9,13,18]. It combines electronic prescribing, barcode medication administration (BCMA), and robotics-based production systems like UDDS [19]. Thus, the combination of BCMA, CDSS and UDDS reduces PEs and medication administration errors (MAE) [20,21]. In turn, the effective digital transformation of hospitals is closely tied to the successful integration of UDDS as a key component of CLMM within the broader digital infrastructure. Within the UDDS process, a CP validates prescriptions entered into the EMS, making necessary adjustments or consults the prescribing physician when required [21,22]. Once validated, UDDS pouches are produced and delivered to the wards, where BCMA ensures accurate administration to the patient [13,23,24]. Nevertheless, IT adoption in Germany’s health sector has been slow, primarily due to insufficient digital infrastructure and a shortage of well-trained personnel to manage the emerging workflows. Consequently, both the implementation and maintenance of UDDS continue to pose substantial resource challenges [25].

At the Helios Group tertiary care teaching hospital in Erfurt, Germany (HK-EF), two blister-pack machines are in operation, each with a processing capacity of up to 400 canisters of medication. These machines support medication supply for 1,680 beds, excluding intensive care unit and paediatric wards [3]. Intensive care units and paediatric wards were excluded from the analysis because they use distinct EMS that is currently incompatible with the UDDS in the study. In addition, their integration was given lower priority due to the relatively small proportion of oral medications prescribed in these patient populations but might be relevant to integrate in the future. In total, 1,862 beds are supplied with medications by the hospital pharmacy at HK-EF, with a subset of up to 1,680 beds being supplied by UDDS. The decision to use combi-dose packaging, where multiple units of the same medication per administration are packed in the same pouch, simplifies dosing and enhances patient safety [26]. Although the clinical advantages of UDDS in minimizing medication errors are well-established, there is limited research into its impact on staffing and pharmacy operating hours [3,13,26,27,28].

During the last decade, the role of a CP has significantly expanded beyond traditional clinical duties [29]. Pharmacists play a crucial intermediary role, connecting UDDS, electronic medication systems, and the wards. CPs are responsible not only for the efficient setup of the system, but also for its ongoing development and maintenance. This ensures seamless medication delivery both in advance and on demand.

Note that solid oral medications classified as carcinogenic, mutagenic, or reprotoxic (CMR) can only be blistered in compliance with strict occupational health and safety regulations. Compliance with these guidelines necessitates additional protective measures and handling procedures that are likely to increase the full-time equivalent (FTE) workload per 100 UDDS beds.

By automating medication management processes, healthcare providers can reallocate time to higher value care activities, including direct patient interaction, clinical decision-making, and personalized care planning [3,17,29]. However, a significant gap remains in the evidence regarding the pharmaceutical personnel workforce required to ensure successful transformation, support, and sustainable maintenance of UDDS [28]. In the context of escalating healthcare costs, rigorous demonstration of the cost-effectiveness of new initiatives is essential for informed decision-making [30]. Thus, the primary objective of this study was to evaluate staffing benchmarks, operational efficiency, and cost optimization for UDSS between 2021 and 2024. Methodologies are presented to quantify the additional CPs and PTAs required to support the expanded responsibilities and to outline necessary personnel resources. In doing so, this study contributes to the broader framework of cost-effectiveness analysis in health economics, particularly within the context of an evolving and transformative healthcare landscape.

## Materials and methods

### Study design and data collection

The study was conducted at HK-EF [3]. In addition to survey data obtained from the Federal Association of German Hospital Pharmacists e. V. (ADKA), operational metrics from HK-EF were extracted from the UDDS machine control system, inventory management software, material management software CGM AMOR and hospital financial databases. Key parameters included monthly blistered tablet volumes, monthly pouch production counts, and staff activity logs detailing task duration as well as machine operating hours.

### Survey of operational hours for UDDS

The survey was disseminated through the mailing list of the Federal Association of German Hospital Pharmacists e. V. (ADKA) targeting hospital professionals [31,32]. ADKA comprises approximately 2,500 voluntary members, the majority of whom are hospital pharmacists (ca. 85%). In 2019, a total of 33 hospitals in Germany were identified as operating UDDS [33], representing 8.9% of the 371 hospital pharmacies nationwide. Of these, 7 institutions participated in the present survey, corresponding to a response rate of ~21%. Participants were asked to provide information on operational hours, the number of hospital beds served by UDDS, staff composition, and shift structures. All responses were anonymized, and results presented in S1 Table.

### Staffing calculation methods

All FTE benchmarks were calculated based on 1,680 beds to ensure consistency with the hospital’s UDDS coverage. To establish precise staffing benchmarks, the study employed the following methodologies:

**Operational hour-based staffing model:** The initial step involved benchmarking baseline staffing requirements according to operational hours at HK-EF against comparable German hospitals using similar UDDS setups. Daily operational hours were categorized into weekday shifts (07:00–17:00) and Saturday shifts (10:00–14:00). The analysis quantified the total hours required to sustain routine operations while adequately meeting service demands.
**Process-based analysis:** To accurately assess labor inputs for each stage of the UDDS workflow, a granular process analysis was conducted. Key tasks include deblistering, prescription validation, blister production, cleaning and quality control. These workstreams were initially timed using manufacturer-provided machine performance metrics, and validated through on-site staff observations. Time requirements for each task were combined and extrapolated to determine the FTEs needed for each operational step.
**Dynamic workflow modeling:** Given the fluctuating demand patterns throughout the day, a dynamic model of staffing requirements was developed. Early-stage tasks, such as deblistering, were identified as requiring minimal staffing during low-demand periods; production activities peaked around 11:00a.m., necessitating higher staffing levels. Demand variations were incorporated into dynamic workflow modeling and allowed flexibly allocated personnel to optimize resource utilization. High-demand periods were addressed by scaling up staffing whereas quieter periods enabled resource conservation.
**Reserve staffing calculations:** To ensure continuity and maintain operational resilience, a reserve staffing model was integrated into the overall framework. A 20% reserve was added to the baseline staffing levels to account for illness, vacations, or emergencies [34,35]. Furthermore, an additional 10% reserve was applied to meet the increased demands in servicing external hospital units.
**Productivity and cost metrics:** To assess UDDS productivity and cost-efficiency, a robust performance metrics framework was implemented focusing on production output, workforce efficiency, and cost optimization over time.
**Blistered tablet/pouch production:** Blistered tablet and pouch production volumes were tracked monthly to evaluate operational throughput and identify trends in longitudinal productivity. Workforce efficiency was assessed by calculating the number of blistered tablets or pouches produced per full-time equivalent (FTE) per month.
**Pouch production costs:** The costs per blister bag was tracked annually. This metric was calculated by dividing the total production costs, excluding the value of the blistered medication, by the number of pouches produced.
**UDDS performance benchmarks:** FTE-to-Bed and FTE-to-10,000 cases ratios were validated through detailed analysis of operational data from HK-EF. All data were corroborated by survey results obtained from other German hospitals.

### Cost–savings trade-off calculations

To assess the economic impact of UDDS implementation, we calculated the trade-off between annual system-related costs and the savings in nursing time. Note that in the economic model, nursing time savings were incorporated as estimated inputs derived from prior studies [3,36], reflecting capacity gains within Germany’s fixed staffing ratios rather than anticipated budgetary reductions.

**Cost calculation:** UDDS-related costs were divided between fixed components (room infrastructure, machine and equipment purchase, and maintenance) and variable components (pharmacy staff FTEs). Total costs amounted to €718,000 in 2022, which is primarily attributable to 7 pharmacy FTEs, with room and machine costs considered fixed. In 2024, following expansion across the entire hospital cluster, costs increased to €840,000. This corresponds to 8.5 FTEs.

**Nursing time savings:** Savings were estimated based on reductions in ward-based medication preparation and dispensing tasks facilitated by UDDS, as previously described [3,36,37] (expressed in nursing FTEs). These data were used as a base and adapted to the number of patient-days in our setting. In 2022, nursing time savings were calculated at approximately €720,000 across 285,000 patient-days. In 2024, the estimated savings exceeded €1.1 million across 435,000 patient-days.

**Trade-off analysis:** Annual savings were then compared with UDDS operating costs to determine the point at which the system became cost-neutral or cost-saving.

### Ethical considerations

The study adhered to institutional and ethical guidelines for research involving operational data. No patient-specific data were collected, thereby ensuring compliance with data protection standards.

### Statistical analysis

Statistical analysis was conducted using GraphPad Prism 10 (version 10.1.2). The normality of data distribution was assessed using the Shapiro-Wilk test [38]. Based on the results of the normality test, a one-way analysis of variance (ANOVA) was used to compare means across multiple groups, identifying statistically significant differences between them. When significant differences were detected through ANOVA, a Dunnett’s post-hoc test was applied, as indicated. The results are presented as mean ± standard error of the mean (SEM). A *p*-value of <0.05 is considered significant.

## Results

### Streamlining UDDS staffing: From survey insights to an operational hour model

A brief survey was conducted in 2019 to gather contextual information on local operational practices as opening hours and staffing practiced in UDDS departments across German hospitals. A total of 7 out of 33 sites responded (~21%). Given the low response rate, the survey findings are presented solely as descriptive context and should not be interpreted as generalizable estimates of system-wide practice. The survey captured information on facility characteristics, workflow descriptions, opening hours, and staffing requirements. The purpose was to establish a reliable metric and identify best practices for optimizing resource allocation and operational efficiency within the HK-EF UDDS production area (S1 Table). It is important to emphasize that the data presented excludes non-UDDS services provided by the hospital pharmacy. The survey revealed that most hospital pharmacies operate the UDDS production areas for at least 9 hours on weekdays, with some extending up to 11 hours. On Saturdays, operating hours typically range between 4–5 hours; UDDS production typically does not take place on Sundays. The findings indicate that the number of hospital beds is not a reliable predictor for determining the operational hours of UDDS production areas. Despite variations in bed capacity among the hospitals, there was minimal correlation between hospital opening hours and the number of beds served. For example, hospitals with fewer than 500 beds operated their UDDS department for 9–11 hours on weekdays, similar to larger hospitals with up to 2,000 beds. This lack of a consistent pattern suggests that factors other than bed capacity — such as institutional policies, staffing availability, or workflow requirements — play a more significant role in determining opening hours.

**Fig 1:**
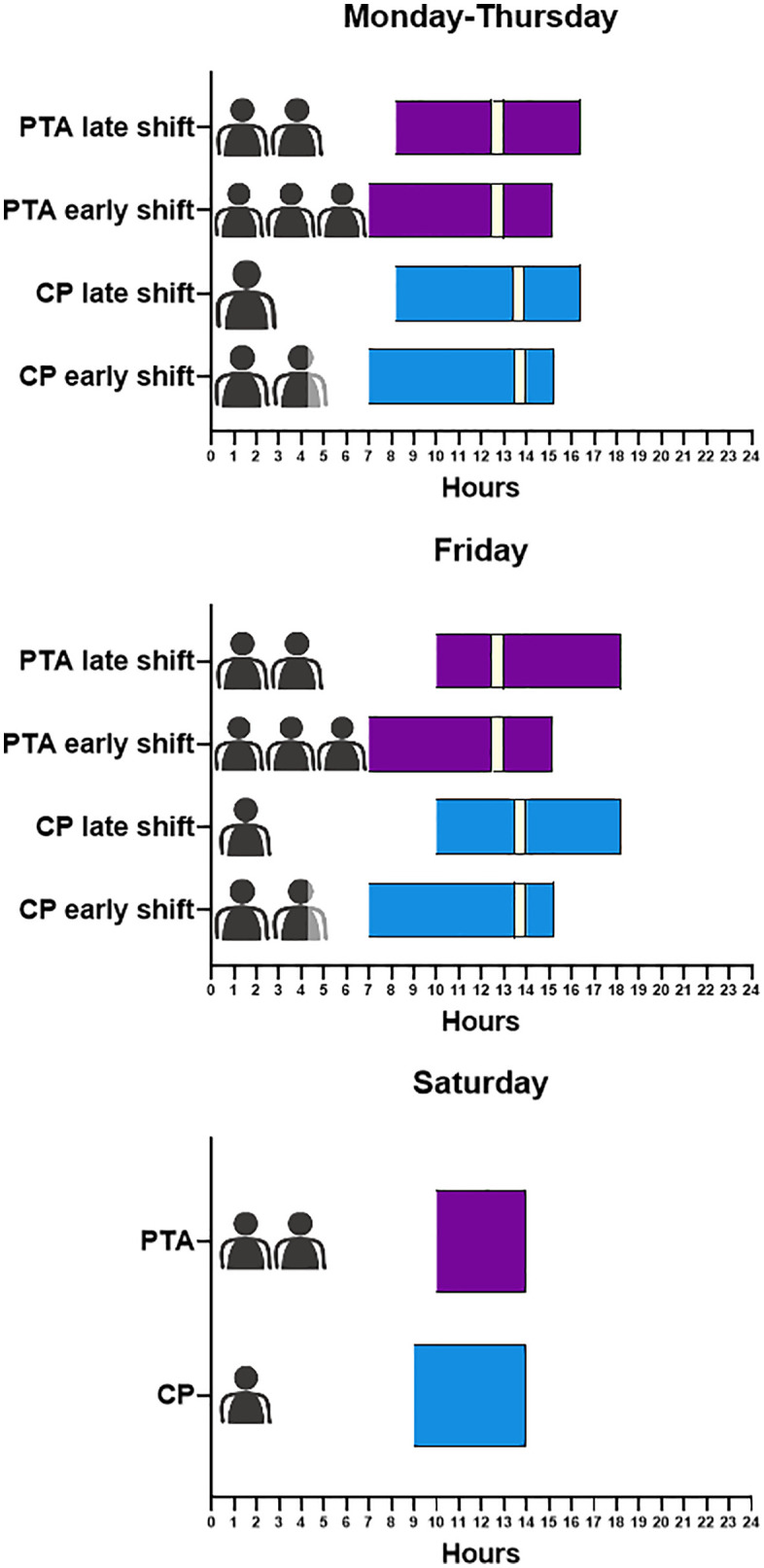
Shift distribution and staffing allocation for PTAs and CPs for 1,680 UDDS beds. The x-axis represents the hours of the day, and the y-axis denotes the shifts for PTA (purple, pharmacy technical assistant), and CP (blue, clinical pharmacist). Break times for both are presented in light yellow. Each shift is accompanied by a pictogram indicating the number of FTEs assigned to it. **(A)** Working hours and FTE allocation from Monday to Thursday, including early and late shifts for both groups. **(B)** Schedule specific to Fridays and **(C)** for Saturdays.

S2 Table presents an initial assessment of staffing requirements for UDDS at HK-EF, detailing the contributions of CPs and PTAs on weekdays (07:00–17:00) and Saturdays (10:00–14:00). The data includes the number of full-time and part-time employees, their respective working hours, and the total hours contributed by each group. Initial staffing estimates are illustrated in Fig 1, which focuses on shift distribution and working hours for both PTAs (purple) and CPs (blue).

From Monday to Thursday, PTAs and CPs cover early and late shifts (Fig 1A). Fridays follow a similar structure with extended working hours (Fig 1B). Saturdays feature reduced hours and simplified shifts, reflecting lower weekend demand (Fig 1C). Tailored to weekly operational needs, Fig 1 shows optimal operating times between 7:00 and 17:00 on weekdays and from 10:00–14:00 on Saturdays. A brief estimate suggests a staffing ratio of approximately ⅓ CPs and ⅔ PTAs. This initial calculation does not account for additional reserves needed to cover staff absences due to illness or vacation [39]. Nevertheless, Fig 1 provides a critical foundation for the following staffing models to ensure sufficient coverage and operational efficiency.

Next, S3 Table incorporates reserve requirements and external bed coverage to provide a more comprehensive and broader calculation of the personnel. The weekly minimum staffing requirement for maintaining operations is calculated at 224.7 hours. Without factoring in potential disruptions like staff absences or increased workload, this figure establishes a clear baseline for smooth functionality. To address those contingencies, a 20% reserve is added [39], raising the total to 269.7 weekly staffing hours. To meet operational demands, seven FTEs are needed, reserve included. If external UDDS beds are serviced, 7.7 FTEs are required, demonstrating the scalability of staffing based on service scope. Staffing efficiency in the literature is often expressed as FTE/100 UDDS beds, providing a standardized metric for resource planning and optimization [3]. All reported FTE values and benchmarks were calculated using the hospital’s UDDS capacity of 1,680 beds, which excludes paediatric and intensive care wards. This standardization provides a reproducible benchmark that other hospitals can use as an initial reference, enhancing comparability across institutions and reflecting economic reality. For HK-EF, with 1,680 UDDS beds, an initial benchmark of 0.45 FTE per 100 UDDS-covered hospital beds was calculated (S3 Table). This calculation aligns staffing requirements with operational needs and maintains flexibility in managing workload variability and capacity changes effectively. However, staffing requirements cannot be determined solely based on operational hours.

### Streamlining UDDS operations: Aligning workload demands with staffing strategies

The production of drugs via UDDS involves multiple steps, each with specific time and labor requirements. To optimize human resource planning, the duration and demands of each step were carefully evaluated. All workstreams are outlined in Table 1, including the respective times required at HK-EF. Preliminary tasks such as warehouse maintenance, deblistering, and cleaning require minimal staffing in the early hours. Around 10:00a.m., prescriptions become available in the EMS, allowing CPs to access and validate them (Workstream VII). During validation, CPs review the physician’s prescriptions, make necessary adjustments, or consult prescribing physicians if necessary. Following validation, the core production process begins around 11:00 AM, which includes operating the UDDS machines, quality control, and packing for delivery to the wards.

**Table 1:** Time and FTE requirements for UDDS workstreams at a teritary care hospital supporting 1,680 beds.

	Workstream	Weekdays [h]	Saturday [h]	Week [h]
I	Warehouse	1.0	0.3	5.3
II	Deblistering	7.7	0.5	39
III	Cleaning UDDS	1.0	0.5	5.5
IV	Operating UDDS	12.5	4.0	66.5
V	Filling tray	4.0	3.0	23
VI	Quality control	6.0	2.0	32
VII	Validation	7.7	2.5	41
VIII	Packing	2.0	0.5	10.5
Total UDDS Process		41.9	13.3	∑222.8
+ 20%	Reserve Requirement	50.3	16.0	267.4
+10%	External UDDS beds	55.3	17.6	294
**FTE**	**1,680 beds**	**7.2**	**2.3**	**7.6**
**FTE / 100 beds**				**0.45**

To ensure operational efficiency, staffing is adjusted dynamically throughout the day. A smaller team handles early tasks; additional personnel are allocated during peak production hours (Fig 1). Staffing levels are reduced as production activity declines later in the day. This strategic allocation prevents overstaffing during periods of low demand and ensures that adequate resources are available during high-demand periods. Based on the calculated duration of each workstream, the personnel requirement is 0.45 FTE per 100 UDDS-covered hospital beds, for a total of 1,680 beds (Table 1). This serves as a precise staffing benchmark for UDDS-driven workstreams. Note that these calculations still represent initial estimates and do not reflect actual productivity levels achieved in practice and/or routine operations.

### Benchmarking UDDS productivity

**Fig 2:**
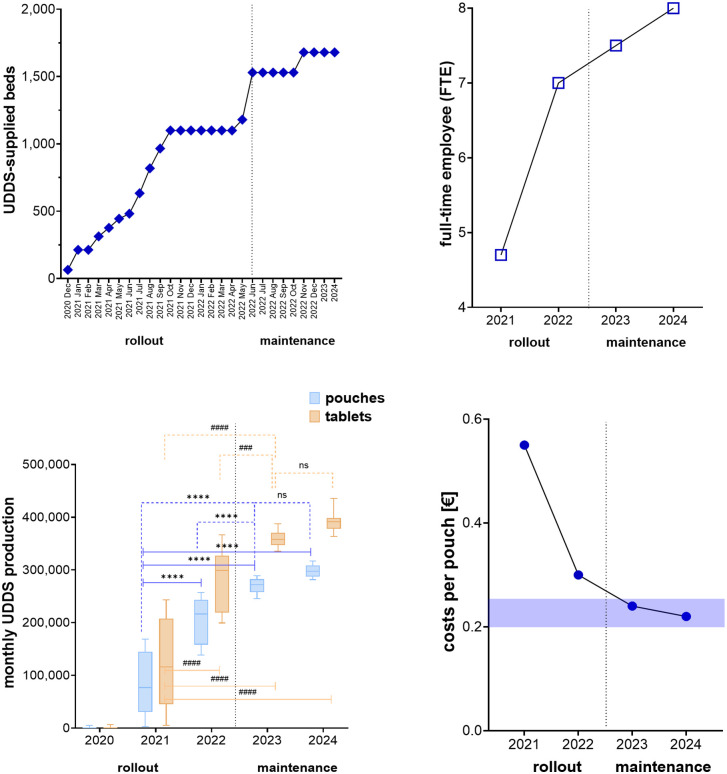
Key performance metrics for unit-dose operations (2020–2024). Performance trends are illustrated across rollout and maintenance phases separated by a dotted line. **(A)** Supplied beds over time: Growth depicted by blue diamonds, with monthly data presented during the rollout phase, transitioning to yearly averages in the maintenance phase. **(B)** FTEs over the years (blue squares). **(C)** Yearly production volumes: Box plots illustrate the annual production of pouches (light blue) and tablets (orange), revealing significant increases from 2022 to 2024 compared to 2021. Dotted lines highlight significant differences relative to 2023, marking the first year of the maintenance phase. *P*-values <0.05 were considered statistically significant, based on ANOVA with Dunnett’s post hoc test. **(D)** Cost per pouch with the median benchmark indicated by a transparent blue area.

To accurately assess and monitor productivity, performance-related benchmarks are necessary to quantify the relationship between staff utilization and output. Key performance metrics for UDDS operations from 2020 to 2024 are highlighted in Fig 2, capturing trends during the rollout and maintenance phases. These trends are essential for understanding operational growth, workforce utilization, productivity improvements, and cost optimization.

Fig 2A illustrates the rapid growth in supplied beds. UDDS coverage increased gradually during the one-year rollout period, ultimately reaching 1,680 beds in the maintenance phase. Note, that the higher total of 1,800 pharmacy-supplied beds at the tertiary care hospital includes paediatric and intensive care units that are not served by UDDS. In detail, during the rollout phase (2020–2022), the number of supplied beds rose sharply, reaching ~1,500 by late 2022. At this point, the pharmacy not only provided UDDS to HK-EF, but also to three smaller associated hospitals (Level I and II hospitals). In the maintenance phase (2023–2024), this number stabilized at 1,680 beds. The transition from rollout to maintenance signifies a shift from a phase of rapid expansion to one of stable, sustained service delivery. As the number of supplied beds influences the number of FTEs, a similar trend was evident here. Fig 2B shows a steep increase in FTEs during the rollout phase (2020–2022) — from ~4.7 in 2021 to 7.5 in 2023 — reflecting higher staffing needs during growth.

In the maintenance phase, FTEs stabilized at around 8, indicating improved efficiency and an optimized workforce. Similarly, the average monthly production of pouches and tablets rose significantly and stabilized at higher levels in the maintenance phase (Fig 2C), reflecting consistent output and workflow management. Finally, costs per pouch — which dropped from €0.55 in 2021 to €0.22 in 2024 (Fig 2D) — can be used as a cost-efficiency benchmark. Overall, Fig 2 provides critical insights into the scaling and stabilization of personnel resources for UDDS.

The rollout phase (2020–2022) is characterized by rapid growth, workforce expansion, and initial cost adjustments. By contrast, the maintenance phase (2023–2024) reflects stabilization in key metrics, including production, staffing, and cost efficiency. These trends indicate the effectiveness of operational strategies in achieving sustainable growth while optimizing resources and maintaining high productivity levels.

**Fig 3:**
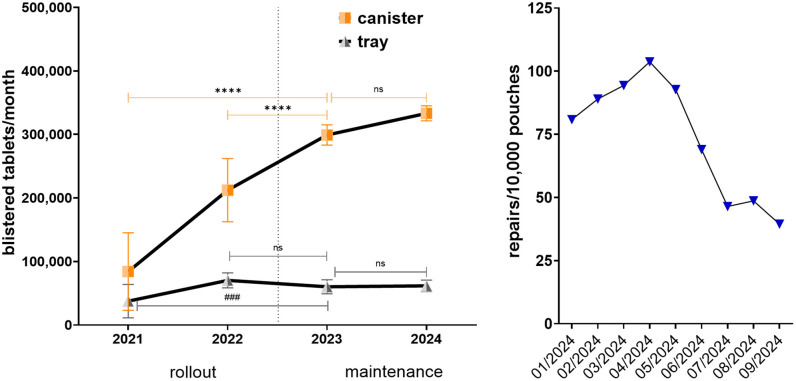
Enhancements in UDDS efficiency. **(A)** Efficiency trends in canister (orange squares) and tray (gray triangles) production show a steady increase in canister output with tray production remaining constant. ANOVA with Dunnett’s post hoc test, using 2023 as the reference point, showed significant increases in canister production efficiency whereas tray production remained unchanged after 2022 (*p* < 0.05). **(B)** Decrease in repairs per 10,000 pouches (dark blue triangles) from January-September 2024.

Clear advancements in UDDS operational efficiency are highlighted in Fig 3. Reduced reliance on tray-based production, the increasing adoption of canister-based supply, and improved system reliability collectively highlights the success of UDDS optimization initiatives during both the rollout and maintenance phases.

In detail, Fig 3A shows a sharp decline in the proportion of tablets produced via trays, dropping below 10% by 2024. This decline reflects a strategic shift toward more efficient production methods and a reduced reliance on tray-based systems. In fact, canister-based production stabilized around 450,000 units per month by 2024, while tray-based production remained consistently low at around 50,000 tablets per month.

These trends demonstrate a clear preference for canister systems due to their higher capacity and efficiency, particularly during the maintenance phase. To ensure quality, all UDDS pouches are inspected by an automated optical control system that verifies both the identity and the quantity of tablets. Detected deviations generate an alert that must be reviewed by a clinical pharmacist. Confirmed errors are corrected manually by opening and repacking the affected pouch; such manual corrections are referred to as repairs.

The number of repairs per 10,000 pouches is shown in Fig 3B, which peaked at over 100 in April 2024, but dropped below 40 by September 2024. A key contributing factor was the change implemented in June 2024: oral cytostatic drugs were no longer routed through the repair line as empty pouches but instead manually filled afterwards. From then on, solid oral medications classified as carcinogenic, mutagenic, or reprotoxic were integrated into the UDDS process and dispensed exclusively via the tray-filling workflow rather than through machine canisters, to minimize contamination risk and maintain safety. This indicates improved system reliability resulting from process optimization and enhanced maintenance.

### Optimizing productivity via strategic resource allocation

**Fig 4:**
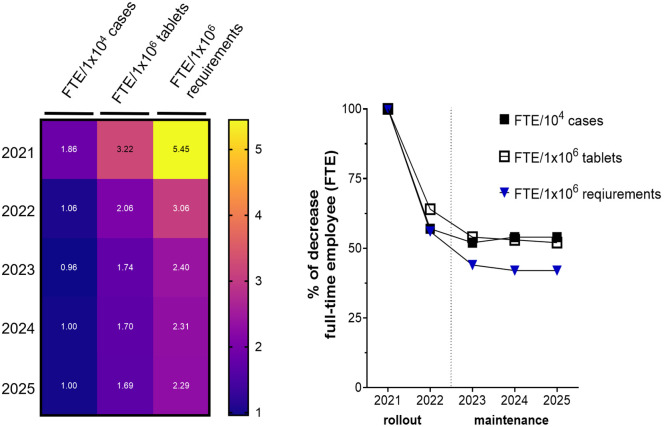
FTE efficiency metrics for UDDS from 2021 to 2025. **(A)** Heat map showing FTE staffing normalized per workload output: per 10⁴ cases, per 10⁶ tablets, and per 10⁶ drug requirements. The color gradient ranges from dark blue (low values, close to 1.0 FTE) to bright yellow (high values, up to 5.5 FTE). **(B)** Percentage decrease in FTE staffing across the three benchmarks: per 10⁴ cases (black squares), per 10⁶ tablets (white squares), and per 10⁶ drug requirements (blue triangle). FTE levels in 2021 served as the reference and were set at 100%. The vertical dotted line indicates the transition from rollout to maintenance.

Shown in Fig 4 are the substantial productivity gains and balanced resource allocation relative to workload output during the rollout (2021–2022) and maintenance (2023–2025) phases of the UDDS implementation. The declining FTE requirements across output metrics, particularly from 2021 to 2025, reflect the significant improvement in operational efficiency. Because real-world data for 2025 are not yet available, a trend-based assessment was conducted to estimate the expected performance of unit-dose systems (Fig 4). These projections offer essential guidance for healthcare institutions, manufacturers, and policymakers as they develop strategies to optimize medication distribution and enhance patient safety in the coming years.

The Fig 4A heat map highlights the FTE needed per 10^4^ cases, 10^6^ tablets, and 10^6^ medication requirements from 2021 to 2025. According to the definition of a “case” in the German Diagnosis Related Groups (DRG) system, a case is one completed, uninterrupted inpatient hospital stay for a patient, used for both statistical and billing purposes in the German healthcare system. If a patient is admitted multiple times in a year, each admission is counted as a separate case. The case numbers were provided by the controlling department of the hospital. Requirements for one drug refer to individual request lines captured in the AMOR inventory management system. A marked reduction in staffing for each benchmark is especially observed from 2021 to 2022, followed by a more gradual decline through 2025. For instance, FTEs per 10⁶ requirements declined from 5.45 in 2021 to 3.06 in 2022, and further reduced to 2.29 by 2025 (Figs 4A and S1). This represents a 58% efficiency gain. Fig 4B quantifies this trend, highlighting the percentage decrease in FTEs. A sharp drop is seen between 2021 and 2022 across all three benchmarks, with relative stabilization during the maintenance phase (2023–2024). FTEs per 10^4^ cases and per 10⁶ tablets show an approximate 50% reduction by 2023, while FTEs per 10⁶ requirements continue to decline gradually through 2025, reflecting ongoing improvements in operational efficiency.

Note that FTE per 10^4^ cases (black squares: Fig 4B and S1 Table) remained stable throughout the study period. Optimal performance was reached as early as 2022 during the rollout phase.

By contrast, the other two metrics stabilized later on during the maintenance phase, beginning in 2023. Overall, Fig 4 shows that the most substantial reduction was achieved in FTE per drug requirement, followed by FTE per blistered tablet; FTE per 10,000 cases reached optimal efficiency earliest. Those results highlight the operational improvements realized during the rollout phase. Further refinements during the maintenance phase led to sustained productivity and balanced resource allocation. S2 Fig complements each finding by outlining the timeline and major milestones associated with the key steps leading to the results.

**Fig 5:**
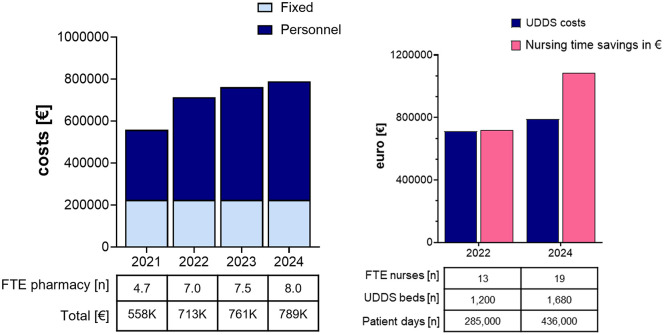
Cost–savings trade-off of UDDS implementation. **(A)** Development of UDDS operating costs between 2021 and 2024. Fixed costs (light blue) include room and UDDS machine implementation costs; variable costs (dark blue) represent pharmacy staff (FTEs). The table below indicates corresponding FTEs and annual costs in thousands of euros. **(B)** Nursing time savings achieved through UDDS implementation. Bars compare UDDS operating costs (blue bar) against the estimated nursing workload reduction expressed as *fictive* cost savings from [3] (pink bar). The table below is indicative of the calculated number of nursing FTEs, and the corresponding number of patient-days, with the associated cost savings modelled at Helios expressed in euros.

The cost of UDDS implementation (equipment, materials, pharmacy staff, and room) is offset by the nursing time savings accrued by outsourcing the medication process, as previously described [3]. In our setting, 2022 costs of €718,000 (7 FTEs) were offset by nursing time savings of €720,000 across 285,000 patient-days. Cluster-wide costs in 2024 came to €840,000 (8.5 FTEs) and were counterbalanced by savings exceeding €1.1 million across 435,000 patient-days. These data indicate that the investment becomes cost-neutral within the first year of full-scale implementation with clear net savings in subsequent years (Fig 5).

## Discussion

Not only are medication errors a significant risk to patient safety, they are also financially costly [18,40]. Each error can lead to additional treatments, extended hospital stays, and legal expenses. Implementing robotics-based dispensing machines like UDDS, which dispense medications in individually labeled doses, minimizes those errors and reduces ADE costs [40–42]. Rather than allocating extra resources for labor-intensive medication preparation tasks, however, healthcare institutions can adjust staffing levels based on real-time patient care needs [3].

Automating medication preparation, packaging, and administration, UDDS reduces cognitive burden and supports standardized workflows, enhancing productivity. They have thus become critical in modern hospital pharmacies, aligning with the digital transformation of healthcare. The current study builds upon prior evidence that demonstrates the benefits of UDDS in improving medication safety and operational efficiency [3,17,43]. Despite that, the impact of UDDS on staffing requirements and workload optimization remained under-explored. Our evaluations solidify the framework for optimizing UDDS personnel planning to achieve maximum efficiency and prevent overstaffing [39,44].

For the HK-EF tertiary care hospital, UDDS staffing requirements were initially determined using a strategic three-step approach. First, based on a survey (S1 Table), weekday operations were planned 7:00–17:00, with no Saturday shifts. This was to limit staffing demands. Next, staffing requirements were assessed, yielding a recommended mix of approximately one-third CP and two-third PTA (Fig 1). However, after integrating 1,000 beds into UDDS in 2021, pre-packaging weekend medications on Friday proved impractical and medically inadvisable: extended hours beyond 7p.m. caused staff fatigue, errors, and dissatisfaction. For example, discrepancies in blister-packed medications frequently required manual corrections by nurses, as unit-dose medications were prepared in advance for UDDS while physicians often modified prescriptions afterwards, e.g., during evening or night shifts. This resulted in mismatches between prepared doses and updated treatment plans. Introducing Saturday shifts resolved those issues by improving workflow and effectively managing weekend admissions. Finally, sub-process durations were analyzed to further refine the estimates (Table 1).

This approach established a standard staffing ratio of 0.40–0.50 FTE per 100 UDDS beds (S2 and S3 Tables), effectively supporting high medication quality at HK-EF. Contrary to assumptions, the number of beds did not correlate with longer operating hours (S1 Table and Fig 1). Efficiency was instead driven by process and workflow optimization (Table 1). At HK-EF, integrating UDDS with EMS ensures optimal drug safety and robust quality assurance measured against internal operational benchmarking. Critical processes such as real-time monitoring, quality controls, and strict regulatory compliance underscore the necessity for continuous quality improvement — particularly as UDDS expands to integrate external hospitals — and were key to minimize errors and maximize system uptime. Such findings align with research highlighting the importance of stringent measures to mitigate risks in automated systems [40,45].

A core outcome of the study was the establishment of a reliable staffing benchmark of 0.45 FTE per 100 UDDS beds, suitable for both rollout and routine operations (Table 1). The HK-EF UDDS department is one of the largest in Germany [46], supplying 1,680 beds at peak efficiency. The described FTE benchmark should be considered in that operational context. Smaller hospitals may require higher FTE-to-output ratios, highlighting the critical role of economies of scale in workforce planning. Furthermore, UDDS staffing is highly influenced by the service level provided by the hospital pharmacy [47]. Aligning with the 80/20 Pareto principle, to expand UDDS coverage beyond the current level of approximately 65% [47] of prescribed medications would require a disproportionate rise in staffing: achieving 80% coverage involves moderate effort whereas reaching 90% or 95% demands significantly more resources [48]. Balancing those trade-offs is crucial to ensuring cost-effectiveness without compromising safety.

Furthermore, the benchmark of 1 FTE per 10^4^ cases proved to be a more practical and dependable standard (Fig 4), especially as it enabled flexible staffing adjustments to accommodate workload fluctuations during periods of peak demand (Fig 1). Previous research has highlighted how adaptive staffing models significantly enhance workflow efficiency and mitigate delays [44]. However, the established FTE benchmarks (Fig 4) alone do not capture the actual productivity of the department. Supplementary metrics, such as pouch costs and blister-packed tablets per FTE, provide deeper insights into work efficiency (Fig 2). Productivity metrics revealed substantial improvements, including a reduction in blister bag costs from €0.55 in 2021 to €0.22 in 2024, meeting the optimal target range of €0.20-€0.25. Personnel optimization was decisive in lowering HK-EF costs per pouch, with €0.58 per blister bag identified by others as a key benchmark [42].

Cost reductions at HK-EF in the study period align with broader research highlighting automation as a key driver of cost efficiency in healthcare settings [30,41]. In our case, blistered tablet production tripled, increasing from 1.4 million to 4.7 million units, without a proportional rise in workforce requirements. Indeed, the FTE required per 10^6^ blister packs fell from 3.2 to 1.7 (Fig 4) — improvements which directly support the effectiveness of UDDS in manual labor reductions and optimizing resource utilization. However, as previously noted, further expansion of UDDS coverage (e.g., > 80%) is likely to yield diminishing returns in efficiency gains [48]. Identifying an optimal balance between expanded coverage and sustainable resource use is still a necessity.

For HK-EF, dynamic staffing adjustments emerged as a pivotal strategy for managing workload variability. Early shift tasks, such as deblistering and warehouse maintenance, required minimal staffing whereas peak production periods necessitated higher personnel engagement (Fig 1 and Table 1). Aligning staffing models with workflow demands ensured effective resource allocation and minimized inefficiencies during low-demand periods while maintaining seamless medication delivery during peak hours. Supplying the hospital wards with UDDS is particularly time-sensitive, especially when serving external hospitals that require precise coordination, logistical planning, robust IT integration and data security. To address those challenges, HK-EF incorporated an additional 10% staffing capacity specifically for external UDDS supply (S2 Table).

The cost–savings analysis in Fig 5 highlights the economic feasibility of UDDS implementation when considered in relation to reductions in nursing workload. While initial investments and ongoing operating costs represent a substantial financial commitment, the costs are effectively balanced by the measurable savings in nursing time [3]. In our setting, the savings achieved through UDDS exceeded system costs within the first year of full rollout, illustrating that the technology can reach cost neutrality rapidly under routine conditions. This finding underscores the relevance of economies of scale: as the number of patient-days increases, the relative benefit of UDDS becomes more pronounced (see 2022 versus 2024 data in Fig 5). Because the cost-neutrality assessment is based on modelled nursing time savings from prior work [3,36,37], these estimates reflect capacity gains rather than actual budget reductions, in line with Germany’s regulated nursing staffing ratios. Thus, the calculated nursing staff savings are only theoretical. In Germany, the number of nurses required is legally defined on a per-bed or per-patient basis, meaning that UDDS implementation does not reduce the absolute number of nurses employed. Instead, the reduction in nursing workload should be interpreted as additional capacity that can be redirected toward direct patient care and other clinical activities, rather than as a true reduction in staff positions.

While the study provides valuable insights, its findings are based on data from a single large tertiary hospital, which may limit generalizability to smaller or less automated facilities. Future research should take account of variations in infrastructure, patient demographics, and personnel resource availability in order to validate the established benchmarks across various healthcare settings. Integration of emerging technologies, such as artificial intelligence and machine learning, likewise offers exciting opportunities for additional staffing models and operational workflow optimizations. Moreover, as observed above, the Pareto principle emphasizes the diminishing returns of extending UDDS coverage yields. This suggests future efforts should prioritize optimizing efficiency within realistic limits rather than striving for maximum coverage at disproportionately high costs.

Efficient staffing is critical for hospital pharmacies, particularly when considered in the context of UDDS implementation for hospitals worldwide. Despite growing adoption of such systems [49], there is a notable lack of standardized benchmarks for personnel requirements [40]. That is a gap in the literature this study has sought to address by presenting a scientifically grounded framework for personnel planning in hospital pharmacies where UDDS has been implemented. A detailed analysis of staffing requirements based on real-world data from the HK-EF large hospital pharmacy — incorporating operating hours, machine runtimes, process workflows, and practical experiences — demonstrates that a recommended staffing ratio of 0.40–0.50 FTE per 100 UDDS beds effectively suffices to maintain high service quality.

Considering our study’s research findings, the FTE per 10^4^ cases and FTE per 10^6^ benchmark requirements directly align staffing needs with the actual volume of managed cases, and are therefore superior to FTE per 100 UDDS beds as a standard measurement. In reflecting live operational demands, those benchmarks thus provide a more precise and dynamic measure of workforce efficiency. Finally, this analysis has also introduced supplementary metrics, such as blister packs per FTE, which offer a deeper understanding of labor efficiency within the UDDS operational context implemented at HK-EF.

## Conclusion

This study underscores the transformative potential of UDDS in revolutionizing hospital pharmacy operations. By emphasizing FTE per 10^4^ cases and per 10^6^ medication requirements as critical new benchmarks, it offers a more accurate and actionable metric for aligning staffing with actual patient care demand, moving beyond traditional, static measures like FTE per bed. This dynamic approach ensures efficient resource utilization, scalability, and cost-effectiveness. The findings present a robust framework for optimizing staffing levels, streamlining workflows, and minimizing costs while maintaining high standards of service quality. Crucially, those benchmarks also function as indicators of patient safety and clinical quality, as more responsive staffing models are linked to reduced medication errors, improved workflow continuity, and enhanced pharmacist engagement in direct patient care [50,51]. Together, these insights support the widespread adoption of UDDS and lay the groundwork for broader innovations in medication management. This will ultimately contribute to safer, more efficient, and sustainable healthcare systems.

## Supporting information

S1 Data Raw data files SmUDo. (XLSX)

S1 Table Survey overview of operating hours for UDDS departments in German hospitals 2019. Results of a survey in 2019 include number of beds and operating hours of UDDS departments on weekdays and Saturdays compared to the teritary care hospital HK-EF.
(DOCX)

S2 Table Initial staffing plan for 1,680 UDDS-covered beds based on operational hours. Overview of staffing distribution for the UDDS department at HK-EF, detailing the number of full-time and part-time employees (FTE), working hours, and total hours per week.
(DOCX)

S3 Table Adjusted staffing plan for UDDS including reserve and external coverage for 1,680 beds. Weekly staffing needs include a 20% reserve and 10% external bed coverage. Calculations show total hours, FTE, and FTE per 100 UDDS beds for efficient resource planning.
(DOCX)

S1 Fig Trends in FTE efficiency (2021–2025). **(A)** Radar plot illustrating FTE needs across three categories: cases (FTE/1x10⁴), tablets (FTE/1x10⁶), and requirements (FTE/1x10⁶). Data trends are shown for 2021 (black dashed line), 2022 (yellow dotted line), 2024 (pink solid line), and for 2025 (blue solid line). The overlayed triangles reflect alignment and balance in operational efficiency among key metrics. **(B)** Trends in FTE/1x10^6^ blistered requirements (blue triangles), FTE/1x10^6^ blistered tablets (orange squares), and FTE 1x10^4^ cases (black circles) from 2021–2024.
(DOCX)

S2 Fig Development in UDDS personnel cost planning (2019–2024). A five-step framework illustrates the evolution of personnel cost planning and benchmarks for UDDS. Key milestones and strategic steps implemented by HK-EF are shown year-by-year, from the initial assessment in 2019 to the establishment of standardized benchmarks and forecasting tools by 2024 for UDDS.
(DOCX)

## Abbreviations

UDDS: Unit-Dose Dispensing System
CLMM: Closed-Loop Medication Management
EMS: Electronic Medication System
CDSS: clinical decision support system
HK-EF: Helios Hospital Erfurt Germany
FTE: full-time equivalent
CP: clinical pharmacist
PTA: pharmaceutical technical assistant, PE, prescription error
ADE: adverse drug event
MAE: medication administration errors.
