# Supplementary material for: SmUDo (Smart Unit-Dose): Redefining efficiency, quality, and staffing strategies for optimized processes
Source: PLoS One. 2026 Jan 16;21(1):e0339381. doi: 10.1371/journal.pone.0339381 (PMC12810781; doi:10.1371/journal.pone.0339381)
Supplement: S1 Table — Results of a survey in 2019 include number of beds and operating hours of UDDS departments on weekdays and Saturdays compared to the teritary care hospital HK-EF. (DOCX) [file pone.0339381.s002.docx]

# **Supporting information**

**SmUDO (Smart Unit-Dose): Redefining efficiency, quality, and staffing strategies for optimized processes**

*Short title: Towards an era of efficiency, safety, and quality in unit-dose*

Jana Gerstmeier, Saskia Herrmann, Annika Demuth, Natalie Vuong, Olaf Kannt and Dominic Fenske

**S1 Table: Survey overview of operating hours for UDDS departments in German hospitals 2019.** Results of a survey in 2019 include number of beds and operating hours of UDDS departments on weekdays and Saturdays compared to the teritary care hospital HK-EF.

**
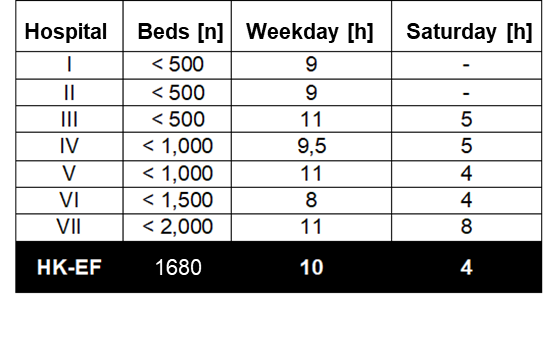
**
