# Supplementary material for: SmUDo (Smart Unit-Dose): Redefining efficiency, quality, and staffing strategies for optimized processes
Source: PLoS One. 2026 Jan 16;21(1):e0339381. doi: 10.1371/journal.pone.0339381 (PMC12810781; doi:10.1371/journal.pone.0339381)

# **Supporting information**

**SmUDO (Smart Unit-Dose): Redefining efficiency, quality, and staffing strategies for optimized processes**

*Short title: Towards an era of efficiency, safety, and quality in unit-dose*

Jana Gerstmeier, Saskia Herrmann, Annika Demuth, Natalie Vuong, Olaf Kannt and Dominic Fenske

**S2 Table: Initial staffing plan for 1,680 UDDS-covered beds based on operational hours.** Overview of staffing distribution for the UDDS department at HK-EF, detailing the number of full-time and part-time employees (FTE), working hours, and total hours per week.


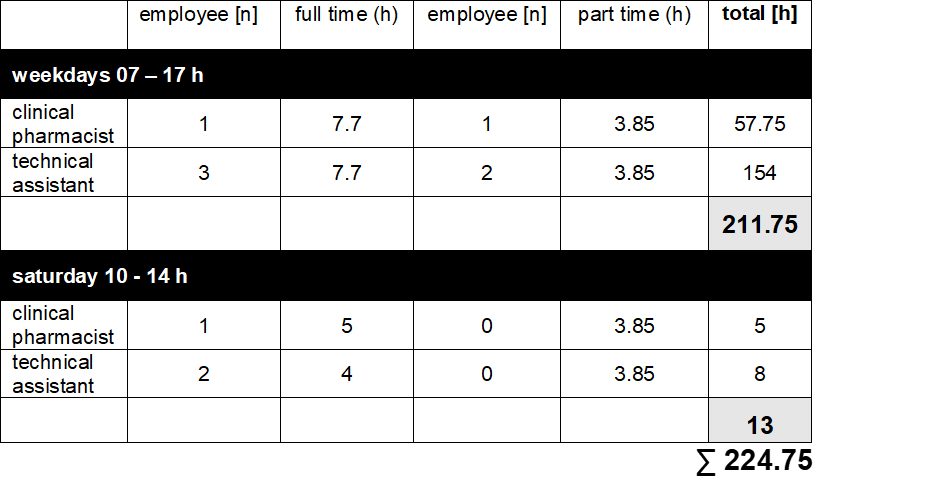

Supplement: S2 Table — Overview of staffing distribution for the UDDS department at HK-EF, detailing the number of full-time and part-time employees (FTE), working hours, and total hours per week. (DOCX) [file pone.0339381.s003.docx]
