# Supplementary material for: SmUDo (Smart Unit-Dose): Redefining efficiency, quality, and staffing strategies for optimized processes
Source: PLoS One. 2026 Jan 16;21(1):e0339381. doi: 10.1371/journal.pone.0339381 (PMC12810781; doi:10.1371/journal.pone.0339381)
Supplement: S3 Table — Weekly staffing needs include a 20% reserve and 10% external bed coverage. Calculations show total hours, FTE, and FTE per 100 UDDS beds for efficient resource planning. (DOCX) [file pone.0339381.s004.docx]

# **Supporting information**

**SmUDO (Smart Unit-Dose): Redefining efficiency, quality, and staffing strategies for optimized processes**

*Short title: Towards an era of efficiency, safety, and quality in unit-dose*

Jana Gerstmeier, Saskia Herrmann, Annika Demuth, Natalie Vuong, Olaf Kannt and Dominic Fenske

**S3 Table: Adjusted staffing plan for UDDS including reserve and external coverage for 1,680 beds.** Weekly staffing needs include a 20% reserve and 10% external bed coverage. Calculations show total hours, FTE, and FTE per 100 UDDS beds for efficient resource planning.

**
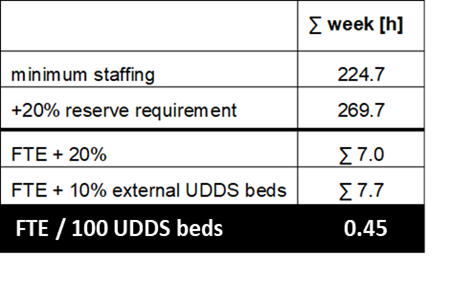
**
