# Supplementary material for: SmUDo (Smart Unit-Dose): Redefining efficiency, quality, and staffing strategies for optimized processes
Source: PLoS One. 2026 Jan 16;21(1):e0339381. doi: 10.1371/journal.pone.0339381 (PMC12810781; doi:10.1371/journal.pone.0339381)
Supplement: S1 Fig — (A) Radar plot illustrating FTE needs across three categories: cases (FTE/1x10⁴), tablets (FTE/1x10⁶), and requirements (FTE/1x10⁶). Data trends are shown for 2021 (black dashed line), 2022 (yellow dotted line), 2024 (pink solid line), and for 2025 (blue solid line). The overlayed triangles reflect alignment and balance in operational efficiency among key metrics. (B) Trends in FTE/1x106 blistered requirements (blue triangles), FTE/1x106 blistered tablets (orange squares), and FTE 1x10^4^ cases (black circles) from 2021–2024. (DOCX) [file pone.0339381.s005.docx]

# **Supporting information**

**SmUDO (Smart Unit-Dose): Redefining efficiency, quality, and staffing strategies for optimized processes**

*Short title: Towards an era of efficiency, safety, and quality in unit-dose*

Jana Gerstmeier, Saskia Herrmann, Annika Demuth, Natalie Vuong, Olaf Kannt and Dominic Fenske

**S1 Figure:**

**A B**


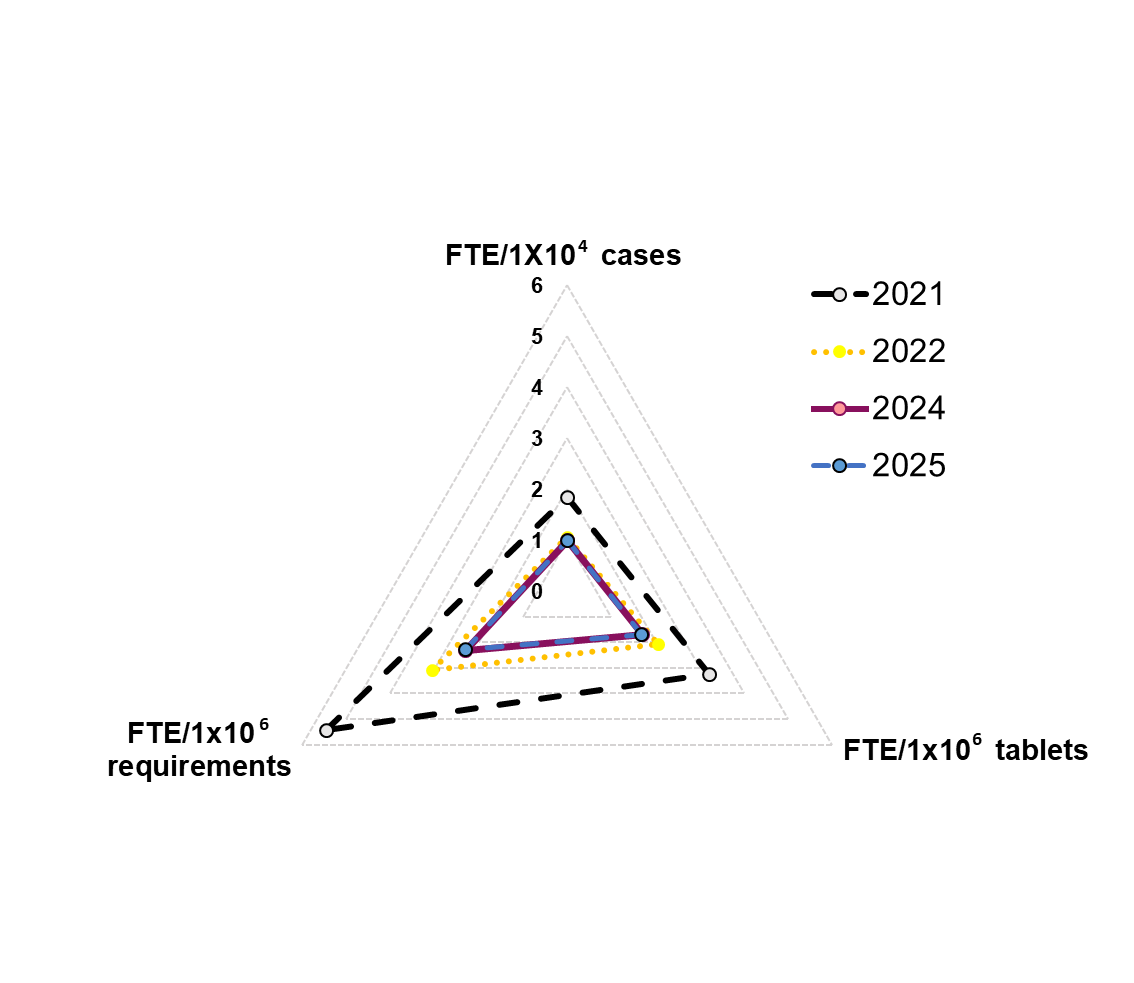


**S1 Fig.:** **Trends in FTE efficiency (2021–2025). (A)** Radar plot illustrating FTE needs across three categories: cases (FTE/1x10⁴), tablets (FTE/1x10⁶), and requirements (FTE/1x10⁶). Data trends are shown for 2021 (black dashed line), 2022 (yellow dotted line), 2024 (pink solid line), and for 2025 (blue solid line). The overlayed triangles reflect alignment and balance in operational efficiency among key metrics. **(B)** Trends in FTE/1x10^6^ blistered requirements (blue triangles), FTE/1x10^6^ blistered tablets (orange squares), and FTE 1x10^4^ cases (black circles) from 2021 - 2024.
