# Supplementary material for: SmUDo (Smart Unit-Dose): Redefining efficiency, quality, and staffing strategies for optimized processes
Source: PLoS One. 2026 Jan 16;21(1):e0339381. doi: 10.1371/journal.pone.0339381 (PMC12810781; doi:10.1371/journal.pone.0339381)
Supplement: S2 Fig — A five-step framework illustrates the evolution of personnel cost planning and benchmarks for UDDS. Key milestones and strategic steps implemented by HK-EF are shown year-by-year, from the initial assessment in 2019 to the establishment of standardized benchmarks and forecasting tools by 2024 for UDDS. (DOCX) [file pone.0339381.s006.docx]

# **Supporting information**

**SmUDO (Smart Unit-Dose): Redefining efficiency, quality, and staffing strategies for optimized processes**

*Short title: Towards an era of efficiency, safety, and quality in unit-dose*

Jana Gerstmeier, Saskia Herrmann, Annika Demuth, Natalie Vuong, Olaf Kannt and Dominic Fenske

**S2 Figure:**

**
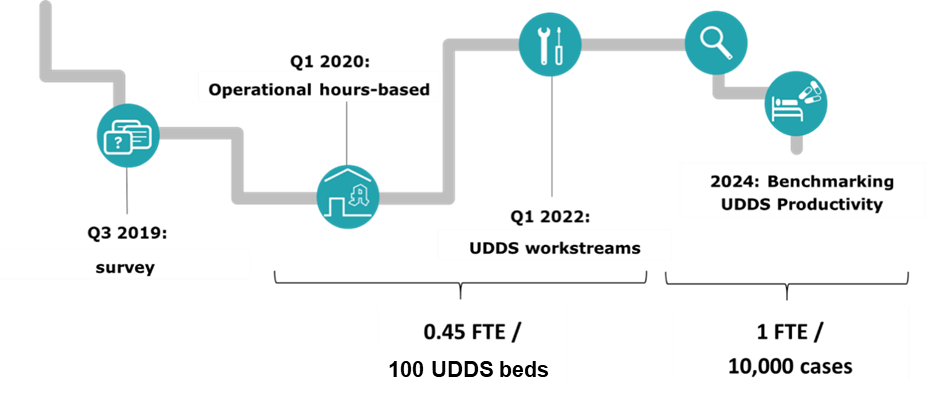
**

**S2 Fig.:** **Development in UDDS personnel cost planning (2019–2024).** A five-step framework illustrating the evolution of personnel cost planning and benchmarks for UDDS. Key milestones and strategic steps implemented by HK-EF are shown year-by-year, from the initial assessment in 2019 to the establishment of standardized benchmarks and forecasting tools by 2024 for UDDS.
